# Husbands’ involvement in antenatal care and its association with women’s utilization of skilled birth attendants in Sidama zone, Ethiopia: a prospective cohort study

**DOI:** 10.1186/s12884-018-1954-3

**Published:** 2018-08-03

**Authors:** Wondwosen Teklesilasie, Wakgari Deressa

**Affiliations:** 1 School of Public and Environmental Health, College of Medicine and Health Sciences, Hawassa University, Hawassa, Ethiopia; 2 Department of Reproductive Health and Health Service Management, School of Public Health, College of Health Sciences, Addis Ababa University, Addis Ababa, Ethiopia; 3 Department of Preventive Medicine, School of Public Health, College of Health Sciences, Addis Ababa University, Addis Ababa, Ethiopia

**Keywords:** Husband, Male involvement, Antenatal care, Skilled birth attendant, Ethiopia

## Abstract

**Background:**

There is limited evidence about husbands’ roles on women’s utilization of skilled maternity care in Ethiopia, a country with low utilization coverage of skilled birth attendants and high maternal mortality. This study examined the association between husbands’ involvement in antenatal care and women’s use of skilled birth attendants in Sidama zone, Southern Ethiopia.

**Methods:**

Using a cohort study design, we followed a random sample of 709 antenatal women until delivery from June 01 to November 30, 2015. Main exposure variable was husband’s involvement in at least one antenatal care visit, and outcome variable was women’s use of skilled attendants during birth. Data were analysed using SPSS software-version20. We computed univariate and bivariate analyses to describe characteristics of the study subjects. A chi-square test with *p*-value < 0.05 level of significance and logistic regression analyses with odds ratio and 95% confidence interval were computed to test homogeneity of the two groups’ baseline characteristics and examine the association between husbands’ involvement in antenatal care and women’s use of skilled attendants during birth. Model assessment of the regression equation was checked using a likelihood ratio test, score test, and Hosmer-Lemeshow goodness-of-fit test.

**Results:**

Women who reported at least one antenatal care visit in which their husbands accompanied them were 6.27 times (95% Confidence interval: 4.2, 9.3) more likely to use skilled birth attendants compared to women attended antenatal care alone.

**Conclusion:**

There was a strong statistically significant association between husbands’ involvement during antenatal care and women’s use of skilled attendants during birth. This implies that woman’s utilization of skilled attendants during birth can be improved by involving their husbands in at least one antenatal care visit.

**Electronic supplementary material:**

The online version of this article (10.1186/s12884-018-1954-3) contains supplementary material, which is available to authorized users.

## Background

Globally, in 2015, approximately eight hundred and thirty women were dying every day due to pregnancy and childbirth complications. Almost all these deaths (99%) occurred in low-resource settings, and more than half occur in sub-Saharan Africa [1–3]. The probability that a 15 year-old girl in sub-Saharan Africa eventually dying from a maternal cause was as high as 1 in 36 compared to 1 in 4900 in developed Region [1, 4].

Ethiopia is one of the six countries that account for 50% of maternal deaths globally [3]. Trends in maternal mortality ratios (MMRs) in Ethiopia, however, showed a significant reduction of maternal deaths from 897 per 100,000 live births in 2000 to 353 per 100,000 live births in 2015 [1]. There is no doubt that the country will need to make huge reductions in MMRs to meet the target of MDG5 and Sustainable Development Goals (SDG3), a transformative new agenda for maternal health towards ending preventable maternal mortality [1]. This will require a great commitment from the government and its partners [5] with community effort.

The challenges of maternal mortality and morbidity in Ethiopia are aggravated by the underutilization of skilled delivery services [6]. The proportion of births with skilled attendants is still very low in Ethiopia [5, 7]. Unskilled persons assist the majority of births at home and only 16% of births are at health facilities in 2014, in Ethiopia [7]. Even though the percentage of facility births continues to be low in Ethiopia, it increased from 5% in 2000 to 16% in 2014 in the last 15 years. In Southern region, 11.7% of the women delivered by SBAs, which is lower than other similar regions in Ethiopia [7].

Ethiopian Federal Ministry of Health (EFMOH), as part of reproductive health (RH) strategies to reduce MMR, had planned to raise the number of deliveries attended by SBAs from 16% in 2006 to 60% by 2015 [5]. This RH strategy did not consider the roles of men in maternal health services. However, the majority of studies in Ethiopia indicates men’s roles as head of the households and key decision-makers of all issues including women’s need for health care services. Moreover, common reasons cited by many studies in Ethiopia for the low utilization of SBAs are focused on the socio-demographic and maternal characteristics, and little has been done to involve male partners in maternal health care services [6, 8, 9].

Evidence from an interventional study in African countries suggest that the three exposure indexes consistently and significantly associated with women’s use of SBAs are husband’s involvement in decision-making, couple’s discussion and planning within the household, and having received counselling on birth preparedness during ANC [8]. Despite that fact, research about the association between husbands’ involvement in ANC visits and women’s utilization of SBAs is limited. Therefore, identifying such association would be important for policy and program planning for maternal health care, specifically to improve women’s utilization of skilled birth attendants. Using a cohort study design, our study sought to determine whether husbands’ involvement in ANC visits is associated with women’s utilization of SBAs.

## Methods

### Design, aim, and setting of the study

This study employed a prospective cohort design to examine the relationship between husbands’ involvement in ANC visits and women’s utilization of SBAs during birth, in Sidama zone, Southern Nations, Nationalities, and People’s Region (SNNPR) of Ethiopia, from June 1, 2015 to November 30, 2015. SNNPR is one of the regions in Ethiopia, with 14 administrative zones and 13 special weredas. One of the most populous zones in the region is Sidama zone. The capital of Sidama zone as well as the region is Hawassa town, which is situated 275 km south of Addis Ababa. The total population size of the zone was 2,966,652; of which 49.5% was females and 5.5% are urban inhabitants [10, 11]. The population density in the zone was 452/km^2^ with an average household size of 4.9 persons. Women of reproductive age (15–49 years) and children under-one-year of age were estimated to be 23 and 3% of the total population, respectively [10]. According to the zonal health department’s report, there are seven primary (district) hospitals, one general hospital (a comprehensive and specialized university hospital), one-hundred and twenty-seven health centres, and five-hundred and twenty-four health posts in the zone. According to 2011 EDHS report, the total fertility rate of the region (SNNPR) was 4.9 children per woman, which was almost similar to the national fertility level of 4.8 children per woman in three years preceding the survey [12]. Skilled ANC, delivery care, and postnatal care (PNC) services (within two days after birth) utilization coverage of the region in the five years preceding the survey were 39, 11.7, and 11.1%, respectively [7].

### Study population and sampling technique

The study was conducted on a random sample of 709 antenatal women who were eligible to be included in this study. The sample-size was estimated by using ‘Power and Sample-Size’ software Program’ (PS version 3.1.2) [13]. The computation was made with 95% confidence level, 5% alpha (α), 80% power (β), and with 1:1 ratio of independent exposed and unexposed groups. We used 90 and 82% of the women attended by SBAs (outcome variable) among women who attended ANC education with their husbands (intervention group) and those who did not attend the services (control group) respectively, from a study in Nepal [14]. The calculated sample-size indicated an appropriate sample size of 638 women. Assumed loss to follow-up was 10%. Hence, total sample of 702 pregnant women were needed to test the hypothesis that women whose husbands were involved during ANC visit are more likely to utilize SBAs during birth compared to women whose husbands were not involved during ANC visit. Then, we applied a random sampling technique to select the study sample. First, we randomly selected eight out of twenty-one weredas in Sidama Zone using a lottery method. Lists of all pregnant women between 24th and 36th weeks of gestational age from June 01 to November 30, 2015 (i.e. 712 women) were prepared using household survey, health institutional records reviews, and by linking them at follow-up schemes with collaborations of the health extension workers (HEWs). Women on the lists were sent invitation letters to participate in the study. Finally, 709 pregnant women were included.

### Inclusion and exclusion criteria

Women with a gestational age (G.A.) between 24th weeks to 36th weeks, living with their husbands for at least a year in the study area and those who initiated ANC service in the selected health institutions before or during the study period were asked to participate. Mothers were excluded if they had complications of pregnancy (high-risk pregnancy confirmed during ANC check-up), being unmarried, and those who were sick and unable to undergo an interview during the study period.

### Definition of exposure and outcome variables

In this study, *husbands’ involvement in antenatal care* (ANC) is the exposure variable- defined as- women who were accompanied by their husbands at least once for ANC visits, and women who attended ANC visits without their husbands were considered as ‘non-exposed’. *Women’s use of skilled birth attendants* (SBAs) was an outcome indicator to evaluate the effect of husbands’ involvement in ANC visits. Women used SBAs - means women were assisted by SBAs during births in health institutions or at home. *Skilled Birth Attendants* (SBAs) – are doctors, nurses, and midwives who have a special training on childbirth practices [8].

### Data collection

Data were collected using a semi-structured follow-up questionnaire, which was prepared for a progressive evaluation of maternal conditions (Additional file 1). Twenty-eight nurses who were working in maternity units and five supervisors collected data after they were trained for three days. Both data collectors and supervisors had a bachelor degree in nursing and a master degree in public health, and had at least one experience in quantitative data collection. The interviews were conducted using the local language (Sidamigna and Amharic) after the questionnaire was pretested for cultural appropriateness and clarity. Data collectors were fluent speakers of both the local and Amharic languages. The questionnaire included three major components: background information of study subjects, past and current obstetric conditions, utilization status of ANC services, husband’s involvement status during ANC visits, and women’s utilization status of SBAs during births.

Data collections were conducted *at three time points.* At the beginning, data collectors approached each potential subject to inquire about their eligibility and interest in volunteering for this study. This inquiry included a description of the study, its importance for maternal health care, responsibilities for participating, and potential benefits of participating.

#### The questions to confirm eligibility included

Information on maternal characteristics such as month of pregnancy, pregnancy intention, initiation of ANC visit, marital status, number of U5 children currently alive, ‘Are you living with your husband or not?’, and ‘Did your husband accompany you for at least one ANC visit in this recent pregnancy?’, and information on women’s background characteristics. All mothers who met the study criteria were asked to participate and give informed consent. Study aids included a client information booklet, writing pad with pen and pencil to calculate expected date of delivery (E.D.D), ‘fundal height’ measurement guide for abdominal palpation were given to data collectors; and telephone numbers of the study team to all HEWs for advice in case of complications or questions regarding follow up visits. During initial assessment, data collectors and supervisors estimated each woman’s date of last ANC check-up (or at 9 months of pregnancy) for next contact.

The *first interviews* with the women were conducted at their last ANC check-up according to the estimated schedule for each woman (or at 9 months of pregnancy). It was to ascertain information on women’s utilization status of ANC services (the number of ANC visits) and the status of their husbands’ involvement in ANC visits (did your husband accompany you for at least one ANC visit? [yes/no]). These included a baseline evaluation and planned visits according to the schedule given for every woman. The *second interviews* were conducted on date of delivery (or within a week after birth) to collect data on women’s place of birth and utilization status of SBAs during birth. If place of births were at health facility, data collectors checked the information available in the delivery care ‘logbook’. Each specific date of interview for every woman was estimated beforehand, based on gestational age by data collectors and supervisors as well as principal investigator. In addition, HEWs at each kebele were assigned to assist data collectors during home visits according to the given schedule. The HEWs in the selected weredas used telephone reminders for upcoming study visits, which was no less frequently than once every month or when there was unscheduled or unexpected childbirth.

### Data processing and analysis

After appropriate coding, data were entered from the completed questionnaires to computer software (SPSS version20) for analysis. Data entry was validated (logic checks including range checks and missing value checks) by visual inspection. Monitoring of the interviews occurred regularly through site visits by supervisors and principal investigator. This included ensuring all questionnaires were completed, few questionnaires were matched randomly with the source of information, and all scheduled and unscheduled visits were documented. Inconsistencies were resolved by contacting the study participants.

We hypothesized that SBA would be higher in the exposed group than in the control group. Study participants were coded as lost to follow-up if the questionnaire on delivery care was not completed. Descriptive statistics were presented mainly as frequency listings and percentages because most of the variables were categorical. For continuous variables, we computed means and standard deviations. We used a chi-square (χ2) goodness-of-fit test with *p*-values < 0.05 for level of significance to test the comparability (homogeneity) of women’s baseline variables (their proportions) in our sample population.

Bivariate logistic regression analyses were computed to examine the association between exposure and outcome variables. Then, we computed multivariate logistic regression analyses to evaluate the independent effects of each predictor variable on the outcome variable by controlling for confounding factors. Odds ratios with 95% confidence intervals (C.I.) were used to evaluate the direction and strength of associations. We checked presence of interaction effects between independent variables, performed an overall model evaluation, statistical test of individual predictors, and goodness-of-fit statistics of the model using SPSS software *version20* (Additional file 2). As women’s demographic and maternal characteristics were a priori expected to have moderation or interaction effects on the relationship between husbands’ involvement and women’s use of SBAs, stratified odds ratios of the exposure and outcome variables were estimated for each independent factor, but are only reported when found to be statistically significant.

## Results

Of 709 study participants, 54% (*n*_1_ = 385) reported at least one ANC visit, in which they were accompanied by their husband whereas 46% (*n*_2_ = 324) reported that they were never accompanied by their husbands. On the first interview dates, women were asked about the total number of ANC visits and whether their husbands’ were involved in at least one or not. This helped to crosscheck their first and second responses, and we took the similar (agreed) responses as a true response to ascertain exposure status. Moreover, 41% (*n* = 143) of the women from the exposed group were observed with their husbands at health facilities for at least one ANC visit during the initial assessment. Fortunately, we did not find variations between the two responses with respect to their husbands’ involvement. During *the second interview sessions*, 664 women (100% of the exposed and 86% of the non-exposed groups) completed their follow-up, yielding a 93.6% response rate. Incomplete follow-up for 45 (6.3%) women from the non-exposed group was due to those who changed their residence with unknown reason even though repeat visits took place.

### Characteristics of study subjects

One hundred and seventy-five (26.4%) respondents were 15 to 24 years of age and 110 (16.6%) were 35 to 49 years of age when their last children were born. (Table 1) Mean age was 28.2 (SD ± 5.43 years). Among these women, 141 (21.2%) had no formal education, 248 (37.3%) had attended primary education, and 176 (26.5%) of the women had attained secondary education. A higher proportion (77.3%, *n* = 513) of the women were ‘housewives’; and the majority (67.6%, *n* = 449) were Protestants while others belonged to Muslim, Orthodox and Catholic religion. (Table 1) The proportion of births at health facility (329; 49.5%) and at home (335; 50.5%) was nearly equal. Similarly, the women’s responses on use of skilled birth attendants (SBAs) and health facility delivery (HFDs) were equal. The two groups of women had nearly even distributions by place of residence, age, level of education, and number of under five children at recent pregnancy (Table 1).

**Table 1:** Baseline characteristics of women in the two groups, in Sidama zone, Southern Ethiopia, 2015

	Was your husband involved at least in one ANC visit?			
Characteristics	Yes, (%) (*n* = 385)	No, (%) (*n* = 279)	Total (*N* = 664)	Χ^2^, (*p*-value)
Residence				
Urban	194 (60.1)	129 (39.9)	323	1.117 (=0.291)
Rural	191 (56.0)	150 (44.0)	341	
Age in year				
15–24	103 (58.9)	72 (41.1)	175	0.090 (=0.956)
25–34	218 (57.5)	161 (42.5)	379	
35–49	64 (58.2)	46 (41.8)	110	
Education level				
Tertiary	62 (62.6)	37 (37.4)	99	3.010 (=0.390)
Secondary	103 (58.5)	73 (41.5)	176	
Primary	134 (54.0)	114 (46.0)	248	
None	86 (61.0)	55 (39.0)	141	
Occupation type				
Government employee	64 (69.6)	28 (30.4)	92	6.159 (=0.046)*
Businesswomen	35 (59.3)	24 (40.7)	59	
Housewife	286 (55.8)	227 (44.2)	513	
Religion				
Protestant	251 (55.9)	198 (44.1)	449	8.908 (=0.031)*
Orthodox	45 (63.4)	26 (36.6)	71	
Catholic	47 (73.4)	17 (26.6)	64	
Muslim	42 (52.5)	38 (47.5)	80	
Initiation of ANC visit				
1st trimester	41 (71.9)	16 (28.1)	57	9.764 (=0.008)**
2nd trimester	231 (60.0)	154 (40.0)	385	
3rd trimester	113 (50.9)	109 (49.1)	222	
Number of ANC visits				
4+	64 (76.2)	20 (23.8)	84	13.087 (< 0.001)***
1 to 3	321 (55.3)	259 (44.7)	580	
Number of under 5 children				
< 1	127 (62.0)	78 (38.0)	205	1.918 (=0.166)
> 1	258 (56.2)	201 (43.8)	459	
Is the pregnancy planned?				
Yes	280 (64.2)	156 (35.8)	436	20.282 (< 0.001)***
No	105 (46.1)	123 (53.9)	228	

***P-value < 0.001, **p-value < 0.01, and *P-value < 0.05, indicate the significance differences of the two groups (women with husbands’ involvement and without involvement by indicated variables)

A higher proportion of the exposed group used SBAs compared to the non-exposed group (67.5%, *n* = 260 versus 24.7%, *n* = 69)). (Table 2) *Variance inflation factors* (VIFs) for each covariate were between ‘1.06 to 1.278’, indicating *no multicollinearity;* since the values are < 5.0 (Additional file 3) [15]. However, the results of stratified analyses showed instability of ORs for ‘initiation of ANC visits during the recent pregnancy’, which has both confounding and modification effect on the relationship between the number of ANC visits and the outcome variable. Due to this reason, the variable ‘initiation of ANC visit’ dropped from the final model.

**Table 2:** Logistic regression analysis of skilled birth attendant utilization by selected characteristics, Sidama Zone, Ethiopia, 2015

Characteristics		Have you received SBAs’ service?			
		Yes, *n* (%)	No, *n* (%)	COR (95%C.I.)	AOR (95%C.I.)
Husband involved in ANC	Yes	260 (67.5)	125 (32.5)	6.33 (4.5, 8.9)*	6.27 (4.2, 9.3)*
	No	69 (24.7)	210 (75.3)	1.00	1.00
Place of residence	Urban	193 (59.8)	130 (40.2)	2.2 (1.6, 3.0)*	1.7 (1.14, 2.5)*
	Rural	136 (39.9)	205 (60.1)	1.00	1.00
Age in year	15–24	100 (57.1)	75 (42.9)	1.9 (1.2, 3.1)*	–
	25–34	184 (48.5)	195 (51.5)	1.4 (0.9, 2.1)	–
	35–49	45 (40.9)	65 (59.1)	1.00	–
Education level	Tertiary	61 (61.6)	38 (38.4)	1.9 (1.14, 3.3)*	1.6 (0.8, 2.9)
	Secondary	102 (58.0)	74 (42.0)	1.7 (1.06, 2.6)*	1.3 (0.7, 2.2)
	Primary	102 (41.1)	146 (58.9)	0.8 (0.5, 1.3)	0.6 (0.4, 1.1)
	None	64 (45.4)	77 (54.6)	1.00	1.00
Occupation type	Government employee	56 (60.9)	36 (39.1)	1.8 (1.2, 2.8)*	–
	Businesswomen	36 (61.0)	23 (39.0)	1.8 (1.05, 3.2)*	–
	Housewife	237 (46.2)	276 (53.8)	1.00	–
Religions	Protestant	207 (46.1)	242 (53.9)	1.5 (0.9–2.5)	1.7 (0.9, 3.1)
	Orthodox	49 (69.0)	22 (31.0)	3.9 (1.9–7.8)*	3.8 (1.7, 8.6)*
	Catholic	44 (68.8)	20 (31.2)	3.8 (1.9–7.8)*	3.4 (1.5, 7.8)*
	Muslim	29 (36.2)	51 (63.8)	1.00	1.00
Planned pregnancy	Yes	262 (60.1)	174 (39.9)	3.6 (2.6–5.1)*	2.5 (1.7, 3.7)*
	No	67 (29.4)	161 (70.6)	1.00	1.00
Number of U5 year children	≤ 1	135 (65.9)	70 (34.1)	2.6 (1.9–3.7)*	2.5 (1.6, 3.8)*
	> 1	194 (42.3)	265 (57.7)	1.00	1.00
Initiation of ANC visit	1st trimester	37 (64.9)	20 (35.1)	3.2 (1.7, 5.8)*	–
	2nd trimester	210 (54.5)	175 (45.5)	2.0 (1.5, 2.9)*	–
	3rd trimester	82 (36.9)	140 (63.1)	1.00	–
Number of ANC visits	4+	66 (78.6)	18 (21.4)	4.4 (2.6–7.6)*	3.3 (1.7, 6.5)*
	1 to 3	263 (45.3)	317 (54.7)	1.00	1.00

*COR* Crude Odds Ratio*, AOR* Adjusted Odds Ratio

*Where 95% C.l. does not include ‘one’, it shows a significant association between the outcome and the factor

The sign”------“indicates the variable was not included in the multivariate analysis

#### Bivariate and multivariate logistic regression analyses

Bivariate analysis showed that husbands’ involvement during ANC visit was statistically significantly associated with women’s use of SBAs during births [COR 6.33, 95% C.I.: 4.5, 8.9]. (Table 2) Regarding women’s background and maternal factors, nine variables were significantly associated with women’s use of SBAs during births, though there was a weak association with women’s age. All independent variables, which had a *p*-value < 0.25 in the bivariate analysis were included in a multivariate regression analysis to ascertain their independent effects on women’s utilization of SBAs during deliveries. (Table 2) On a multivariate analysis, husbands’ involvement during ANC visits and women’s use of SBAs during birth showed a strong significant association [AOR 6.27, 95% C.I.: 4.2, 9.3] (Table 2).

There was no homogeneity of the odds ratios for the relationship between ‘*husbands’ involvement during ANC visit’* and ‘*women’s use of SBA’* across categories of place of residence and women’s age (as layer variables). (Table 3) However, across categories of *women’s education* (as layer variable), a significance value of homogeneity test was greater than 0.10, indicating homogeneous odds ratios among categories. (Table 3).

**Table 3:** Homogeneity tests for Odds Ratio of factors (layer variable), in Sidama zone, Southern Ethiopia, 2015

Layer variable (by categories)		Chi-Squared	df	Asymp. Sig. (2-sided)
Place of residence (2 categories.)	Breslow-Day	9.808	1	0.002
	Tarone’s	9.806	1	0.002
Women’s age (3 categories)	Breslow-Day	21.613	2	0.000
	Tarone’s	21.455	2	0.000
Women’s education level (4 categories)	Breslow-Day	4.114	3	0.249*
	Tarone’s	4.112	3	0.250*

*p-value > 0.10, indicating homogeneity of the odds ratios among categories of a particular variable

Among women’s background and maternal factors, place of residence, religion, number of under five children, and number of ANC visits attended by women during the recent pregnancy were found to be significant predictors for women’s use of SBAs during births, after controlling for confounding variables. Women with one or no U5 children during the recent pregnancy were 2.5 times more likely to use SBAs during births compared to women with more than one U5 children during the recent births [AOR 2.5, 95%C.I.: 1.6, 3.8]. Similarly, urban women were 1.7 times more likely to use SBAs during births compared to rural women [AOR 1.7, 95% C.I.: 1.14, 2.5]. (Table 2) Women who belong to Orthodox [AOR 3.8, 95% C.I.: 1.7, 8.6] and Catholic [AOR 3.4, 95% C.I.: 1.5, 7.8] religions were 3.8 and 3.4 times more likely to use SBAs during births compared to Muslim women. (Table 2).

The odds of using SBAs during birth for women with planned pregnancies were 2.5 more times as likely as women with unplanned pregnancies [AOR 2.5, 95%C.I.: 1.7, 3.7]. Women who attended four or more ANC visits during recent pregnancies were 3.3 times more likely to use SBAs during birth compared to women who attended less than or equal to three ANC visits [AOR 3.3, 95% C.I.: 1.7, 6.5]. In addition, there was a positive relationship between number of ANC visits and odds of using SBAs during births. (Table 2).

## Discussion

A higher proportion of women from the exposed group attended skilled assistance during birth compared to the non-exposed group. Husbands’ involvement in their wives’ ANC visits was an important predictor found to be strongly and significantly associated with women’s use of SBAs during birth. This is consistent with other studies in Africa including Ethiopia, and Asia [16–20]. This could be due to a man involved in ANC who is more likely to discuss and jointly decide on his wife’s place of birth than an uninvolved man. This is supported by studies in Ethiopia and Uganda [16, 21]. Male partners’ concern and the presence of open discussion between partners may help a woman to use SBAs during birth. Similarly, our finding reinforces studies in Uganda [18] and Bangladesh [19] showing that women were more likely to have better outcomes when their husbands were directly involved in maternal health care by attending ANC visits and by supporting them during pregnancy. This implies that the influences from other people (especially, partners) are important determinants of women’s delivery place [8, 17, 20].

There were variations in the influence of husbands’ involvement during ANC visits on women’s use of SBAs across categories of residence and women’s age, although it did not differ with respect of categories of women’s education levels. This implies that women’s odds of using SBAs during births may be increased by targeting husbands of both rural women and young women (age 15–25 years old) to be involved in their wives’ ANC visits. Women who reside in urban places were more likely to use SBAs during births compared to rural women. This is consistent with other studies in Ethiopia and other African countries [6, 9, 22]. A likely explanation for this relationship is that urban women are more accessible for health information through either mass media or other sources. Moreover, as in most sub-Saharan African countries, urban women in Ethiopia tend to benefit from relatively easy access to maternal health services [22]. However, currently, the Ethiopian government has tried to reach rural communities with health information through HEWs as well as some electronic media like television and mobile phones.

Women who belong to Orthodox and Catholic religions were more likely to use SBAs during birth than women belong to Muslim religion. This is consistent with other studies in Ethiopia, Asia, and Kenya [6, 22–29]. A previous study in Ethiopia, however, did not find a significant association between religion and SBAs utilization [9]. This difference might be due to the difference in study context, study design and sample size. Further studies are needed to ascertain the discrepancies with respect to religion.

The relationship between level of education and women’s use of SBAs during birth did not attain statistical significance. It is similar with another study in Ethiopia that showed women’s education is a weak predictor of the use of skilled assistance at birth [6]. Other studies in Ethiopia [9, 11, 22, 28], however, documented that women’s education is a major factor influencing maternal health care utilization, but these addressed maternity care services in general and utilization of SBAs not as a separate issue. It is commonly believed that education serves as proxy for information and knowledge of available health care services [30]. Moreover, education enhances level of women’s autonomy and increases decision-making power that results in improved freedom to make decisions including the use of maternal health care [31]. A study in Tanzania reported that educated women were more likely to make decisions to use assistance from medical personnel at birth themselves compared to their uneducated counterparts [32]. Similarly, studies in Bangladesh [33] and Turkey [34] found that women’s education was a strong determinant of the use of skilled assistance at birth. Possible explanations for the differences may be, first, due to differences in study designs and sample sizes; second, it could be due to an effect modification between women’s education levels and our main exposure variable in the association with the outcome variable. So, how women’s education levels influence SBAs utilization with and without the influence of husbands require further study.

The association of women’s use of SBAs during birth with the number of ANC visits, the number of under five children, and the pregnancy intention are consistent with three studies in Ethiopia [6, 35, 36], studies in Kenya [17, 37], Tanzania [38] and Nepal [39]. It is, however, not only the quantity but also the quality of ANC that influences care seeking during birth [8]. Although this study did not address quality of ANC services, a study in Africa showed that women with the highest focused ANC Index scores (care involved both quantity and quality) were three times more likely to deliver in health facilities than women with the lowest scores. This illustrates that even in an area where women have a strong preference to use traditional birth attendants for delivery, quality and quantity of ANC can have a major impact on care-seeking [8]. Women who had one or no under-five children were more likely to use SBAs during birth than those with more than one. Similarly, women whose pregnancy was planned were more likely to use SBAs during birth compared to women with unplanned pregnancy. This is consistent with studies in South and South-East Ethiopia [6, 35] and Kenya [17, 37]. Women with two or more under five children may prefer to deliver at home because of experiences from previous birth. Parents with fewer children lack experience and thus may seek more easily professional care [9, 28]. In addition, the first deliveries tend to be more difficult and that may motivate women for institutional delivery [6, 28].

### Study limitation

Loss of follow up was a limitation in this cohort study. Respondents often changed their place of residence during follow-up time. To manage this, we tried repeated visits during data collection periods. To examine the effect of the lost follow-up cases, we computed an intent-to-treat analysis and there was no significant difference on the outcomes. The other potential biases in this study may be social desirability bias in favour of their husbands’ involvement in ANC service. To reduce this, we tried to interview each woman in a separate room by explaining the benefits of their honest responses for improvement of maternal health services, and we used repeated interviews (before and after delivery) to ensure the response was genuine. Further limitation of this study is data on service quality were not included. Also data on household income were incomplete, since most respondents did not volunteer to respond to such questions due to unknown reasons. This may have influenced both husbands’ involvement and women’s use of skilled services.

### Strengths of the study

The strengths of this study were, first, a cohort design and an adequate sample size. Secondly, the observations of up to 41% of women being accompanied by their husbands lend credence to the findings from the interviews. Moreover, it is the first study in the region to determine the relationship between husbands’ involvement in ANC services and women’s utilization of skilled birth attendants during delivery.

## Conclusions and recommendations

The strong relationship between husband’s involvement during ANC visits and women’s use of SBAs during birth implies that raising awareness about husbands’ involvement during ANC visits through mass media, religious leaders and community elders should be given due attention, especially in regions and areas with contextual similarities. Besides that, there is a need to promote husbands’ involvement in their wives’ ANC visits by targeting large proportions of rural and young women. Further, it is necessary to provide information and advice on the frequency of ANC visits, at least four visits per pregnancy through contextual based couple’s ANC counselling sessions. Therefore, there is a need for planners of maternal health programs to develop contextual-based approaches that promote husbands’ involvement in maternal health care at Wereda, Zone and Regional levels. Our findings also strongly recommend a policy to mainstream male involvement in routine maternal health care. Such policies should address husbands’ roles and constraints, as well as an educational component to sensitize husbands to the benefits of their involvement in pregnancy care and outcomes. Based on our findings, we also strongly recommend health institutions to start well-organized ANC couples’ counselling sessions at least once after the first ANC visit of every pregnant woman based on the women’s consent. Antenatal care couple-counselling sessions should address the benefits of skilled maternal health care in general; and the benefits of repeated ANC visits and postpartum services in particular. For men, such efforts could help to deflate the assumption that maternal health care is an exclusively women’s concern.

## Additional files

Additional file 1: Participants’ Information Sheet & Consent-form. (PDF 219 kb)

Additional file 2: SPSS Output-logistic regression table. The data described the output of a multivariate analysis of selected predictors for women’s utilization of skilled birth attendants; and it is the best-fitted model selected from six models constructed by forward log-likelihood methods in SPSS. (PDF 176 kb)

Additional file 3: Multicollinearity statistics output table. The data described the multicollinearity statistics output- i.e. the tolerance and Variance Inflation Factor- of selected independent variables. (PDF 168 kb)

Additional file 4: English version Interview Questionnaire. (PDF 271 kb)

## Abbreviations

ANC: Antenatal Care
AOR: Adjusted Odds Ratio
CI: Confidence Interval
COR: Crude Odds Ratio
E.D.D.: Expected Date of Delivery;
EDHS: Ethiopia Demographic and Health Survey
EFMOH: Ethiopia Federal Ministry Of Health
G.A.: Gestational Age
HEW: Health Extension Worker
MMR: Maternal Mortality Rate/Ratio
OR: Odds Ratio
PNC: Postnatal Care
RH: Reproductive Health
SBA: Skilled Birth Attendant
SDG: Sustainable Development Goals
SNNPR: Southern Nation Nationality and People’s Region
SPSS: Statistical Software Package for Social Science
VIF: Variance Inflation Factor
