# Supplementary material for: Husbands’ involvement in antenatal care and its association with women’s utilization of skilled birth attendants in Sidama zone, Ethiopia: a prospective cohort study
Source: BMC Pregnancy Childbirth. 2018 Aug 3;18:315. doi: 10.1186/s12884-018-1954-3 (PMC6091095; doi:10.1186/s12884-018-1954-3)
Supplement: Supplementary file 1 — Participants’ Information Sheet & Consent-form. (PDF 219 kb) [file 12884_2018_1954_MOESM1_ESM.pdf]

Additional file 1: Participant's Information Sheet and Informed Written Consent Form

A. Participant's Information Sheet

Title of the project: Husbands' involvement during antenatal care visits and its association with women's utilization of skilled birth attendants in Sidama Zone, Southern Ethiopia.
Ethiopia: A Prospective Cohort Study

Principal Investigator: Wondwossen T/silasie
Co-investigator: Dr. Wakgari Deressa

Coordinating office: Addis Ababa University, School of Public Health

Introduction: Maternal health is one of the major concerns of public health organizations, policy makers, and researchers throughout the world, particularly in developing countries. This is because of high number of pregnancy related health complications and death of women. One major reason is the non-utilization of available maternal health services. Among the factors for low utilization of maternal-health services by women could also be low husbands involvement in these services.

Purpose: This study aimed to determine the association between husbands' involvement during antenatal care visits and women's utilization of skilled birth attendants in Sidama Zone.

Procedure and Participation: we will use an analytic study designs to follow eligible ANC mothers.
The expected duration of the participant's contact with the interviewer will be not more than fifty minutes. You asked to participate in this research because the trustful information, which you will provide, is important for the understanding of the proposed subject matter. The interview will include your view and experiences related to husbands' involvement and your utilization of maternal health care services.

Confidentiality: to establish secured safeguards of the confidentiality of research data, the PI will use codes during data collection period instead of using names. The original data will be locked in cabinets until the data analysis carryout and no person shall access except the PI and the co-investigator for data checking and cleaning purpose. The use of information for any purpose other than that to which participants consented is unethical to the participants. The information you provide is not disclosed in the way it identified your personal characteristics and privacy. After the final work is approved by the school of public health and academic commission and university senate, the original data questionnaire will be burned in secure manner.

Benefit: The research does not have a short term financial, health care and capacity building benefit to the research participant as an individual or as a group. But in the long run it will help the concerned organization and policy makers to have a policy consideration and direction, and formulation of strategy and design of husbands' involvement in maternal health services based on the recommendations and the findings.

Risk: The proposed research does not have any inhumane treatment of research participants and any physical harm, social discrimination, psychological trauma and economic loss.

Inducement, incentive, and Compensation: The study process will have not any form of inducement, coercion and the study does not bring any risks that incur compensation.

Results Dissemination: The researcher is responsible for dissemination of findings moreover fully accountable to provide feedback to the health institutions and to the policy makers. Maximum effort will be done to publish the finding in scientific reputable journal.

Freedom to withdraw: If you want to participant in the study, you have full right to with draw from the study any time you wish. This would have no effect at all on your health benefit or other administrative effect that you get from the health institutions as routine moreover no body will enforce you to explain the reason of withdrawal.

Person to Contact: The participant has the right to ask information that is not clear about the research context and content before and or during the research work. You can contact the principal investigator and his advisor. Moreover, this research undergone ethical reviewed and approved by Addis Ababa University College of Health Sciences IRB. The main task of this board is to make sure that the ethical principles is adhered or not and the research participants are protected from harm.

If you want more information and check about this project, you can contact the following people:

Addis Ababa University College of Health Sciences IRB Secretary Office Tel. +251-115512876

School of Public Health: Office phone: +251-11-515 77 01 P.O.Box: 6850 Addis Ababa, Ethiopia

Principal Investigator name and address: Wondwossen T/silasie, Tel: +251-911424432;

E-mail: wondeti@yahoo.com

B. Informed written consent form

Title of the project: Husbands' involvement during antenatal care visits and its association with women's utilization of skilled birth attendants in Sidama Zone, Southern Ethiopia: A Prospective Cohort Study

I have been well aware of that this research undertaking is supported and coordinated by AAU School of Public Health and the designate principal investigator is Wondwossen T/silasie. I have been fully informed in the language I understand about the research project objectives that are to understand about husbands' involvement and its influence on women's utilization of maternal health care services.

I have been informed that all the information I shall provide to the interviewer will be kept confidential. I understood that the research has no any risk and no composition. I also knew that I have the right to withhold information, skip questions to answer or to withdraw from the study any time I have acquainted nobody will impose me to explain the reason of withdrawal. It is also enlighten there would have no effect at all in my health benefit or other administrative effect that I get from the hospital. I have assured that the right to ask information that is not clear about the research before and or during the research work and to contact:

Addis Ababa University College of Health Sciences IRB Secretary Office Tel. +251-115512876

School of Public Health: Office phone: +251-11-515 77 01 P.O.Box: 6850

Principal Investigator name and address: Wondwossen T/silasie, Tel: +251-911424432;

E-mail: wondeti@yahoo.com

I have read this form, or it has been read to me in the language I comprehend and understood the condition stated above, therefore, I am willing and confirm my participation by signing the consent.

Name of the participant ______________________

Agreed to participate in the study: Mark 'yes' if agreed, or 'No' if not agreed)

If 'yes', signature _____________

Name of witness signature ____________ (Data collector, supervisor, any third person)

Signature ______________________

Date _______________________
