# Supplementary material for: Husbands’ involvement in antenatal care and its association with women’s utilization of skilled birth attendants in Sidama zone, Ethiopia: a prospective cohort study
Source: BMC Pregnancy Childbirth. 2018 Aug 3;18:315. doi: 10.1186/s12884-018-1954-3 (PMC6091095; doi:10.1186/s12884-018-1954-3)
Supplement: Supplementary file 2 — SPSS Output-logistic regression table. The data described the output of a multivariate analysis of selected predictors for women’s utilization of skilled birth attendants; and it is the best-fitted model selected from six models constructed by forward log-likelihood methods in SPSS. (PDF 176 kb) [file 12884_2018_1954_MOESM2_ESM.pdf]

Additional file 2. SPSS output: logistic regression, Model evaluation and Goodness-of-fit test

Predictor	B	SE.B	Wald's X^2	df.	P-value	e^B (OR)	95% CI.
Constant	*-2.969*	*0.405*	*53.850*	*1*	*0.000*	*0.051*	
Husband involvement in ANC (1= yes, 0= no)	1.836	0.199	84.923	1	0.000	6.273	4.2, 9.3*
Place of residence (1= urban, 0= rural)	0.533	0.205	6.739	1	0.009	1.704	1.1, 2.5*
Education (0= no formal education)			11.260	3	0.010		
Education (1) primary	-0.445	0.263	2.877	1	0.090	0.641	0.4, 1.1
Education (2) secondary	0.228	0.278	0.672	1	0.412	1.256	0.7, 2.2
Education (3) tertiary	0.444	0.325	1.864	1	0.172	1.559	0.8, 3.0
Religion (0= Muslim)			14.178	3	0.003		
Religion (1) Protestant	0.546	0.305	3.203	1	0.073	1.726	0.9, 3.1
Religion(2) Catholic	1.230	0.424	8.407	1	0.004	3.421	1.5, 7.8*
Religion(3) Orthodox	1.336	0.417	10.250	1	0.001	3.804	1.7, 8.6*
Plan of pregnancy (1= yes, 0= no)	0.908	0.206	19.498	1	0.000	2.480	1.7, 3.7*
Number of under 5 years children	0.912	0.220	17.147	1	0.000	2.490	1.6, 3.8*
Number of ANC visits	1.197	0.346	11.999	1	0.001	3.310	1.7, 6.5*

Tests	X^2	df.	P-value
Overall model evaluation			
▪ Likelihood ratio test	241.306	11	0.000
▪ Score test	211.066	17	0.000
Goodness-of-fit test			
▪ Hosmer & Lemeshow	15.274	8	0.054

[+]*Cox and Snell R^2 = 0.305, and Nagelkerke R^2 = 0.406*

**p-value < 0.05, indicates the presence of significant association between the particular factor and use of skilled birth attendants*
