# Supplementary material for: Husbands’ involvement in antenatal care and its association with women’s utilization of skilled birth attendants in Sidama zone, Ethiopia: a prospective cohort study
Source: BMC Pregnancy Childbirth. 2018 Aug 3;18:315. doi: 10.1186/s12884-018-1954-3 (PMC6091095; doi:10.1186/s12884-018-1954-3)
Supplement: Supplementary file 3 — Multicollinearity statistics output table. The data described the multicollinearity statistics output- i.e. the tolerance and Variance Inflation Factor- of selected independent variables. (PDF 168 kb) [file 12884_2018_1954_MOESM3_ESM.pdf]

Additional file 3. Multicollinearity statistics: Tolerance and Variance Inflation Factors, by selected independent variables

Model	Unstandardized Coefficients		Standardized Coefficients	t	Sig.	Collinearity Statistics	
	B	Std. Error	Beta			Tolerance	VIF[a]
(Constant)	.043	.053		.819	.413		
Husband's involvement	.360	.034	.355	10.530	.000	.943	1.060*
Place of resident	.106	.036	.106	2.948	.003	.829	1.207
Age Category	-.043	.036	-.042	-1.185	.236	.847	1.180
Educational level	.087	.037	.086	2.384	.017	.830	1.205
Religion	-.048	.036	-.045	-1.320	.187	.921	1.086
Women occupation type	.025	.044	.021	.584	.559	.805	1.243
Number of u5 child	.150	.040	.139	3.750	.000	.783	1.278**
Planned pregnancy	.170	.037	.162	4.548	.000	.851	1.175
Time of 1st ANC visit	.046	.037	.043	1.231	.219	.876	1.142
Number of ANC visits	.162	.053	.107	3.069	.002	.877	1.140

Dependent Variable: use of skilled birth attendants

[a]VIF: Variance Inflation Factor

* *Minimum VIF value, ***Maximum VIF value*
