# Supplementary material for: Husbands’ involvement in antenatal care and its association with women’s utilization of skilled birth attendants in Sidama zone, Ethiopia: a prospective cohort study
Source: BMC Pregnancy Childbirth. 2018 Aug 3;18:315. doi: 10.1186/s12884-018-1954-3 (PMC6091095; doi:10.1186/s12884-018-1954-3)
Supplement: Supplementary file 4 — English version Interview Questionnaire. (PDF 271 kb) [file 12884_2018_1954_MOESM4_ESM.pdf]

Additional file 4: Interview questionnaire for women participants

Households Identification

Questionnaire code -------------------- Woreda/ Town Administration -----------------

Place of Usual residence: (1) Urban (2) Rural

Kebele ----------------------- House number ----------------

Instruction: – please circle the responses for questions with alternatives and write for open-ended questions on the space provided.

PHASE I - IDENTIFICATION OF STUDY PARTICIPANTS			
**1. Socio-demographic and economic Information**			
**No**	**Questions**	**Choices**	**Code skip**
101	Age (In completed years)	………… Years	
102	Educational level	1. Above grade 12<br>2. Grade 11-12<br>3. Grade 7-10<br>4. Grade 0-6<br>5. Able to read and write<br>6. Unable to read and write	
103	Religion	1. Orthodox<br>2. Catholic<br>3. Protestant<br>4. Muslim<br>5. If other, specify __________	
104	Ethnicity	1. Sidama 5. Gurage<br>2. Wolita 6. Amhara<br>3. Gedio 7. Oromo<br>4. Kembata 8. Tigray.<br>9. If other specify _____	
105	Occupation type	1. House wife<br>2. Farmer<br>3. Government employee<br>4. Privately owned business<br>5. NGO employee<br>6. If other specify __________	
106	Age of your husband (In completed years)	………… Years	
107	Education level of your husband	1. Above grade 12<br>2. Grade 11-12<br>3. Grade 7-10<br>4. Grade 0-6<br>5. Able to read and write<br>6. Unable to read and write	

108	Occupation type of your husband	1. Farmer<br>2. Government employee<br>3. Privately owned business<br>4. NGO employee<br>5. If other specify __________	
109	Household size	-------------	
110	How many Under 5 year children do you have, currently?	-------------	
111	How much is your monthly income? (in ETB)	……………	

**2. Obstetric Information**			
**No**	**Questions**	**Choices**	**Remark**
201	Number of deliveries? (Including those who died, if any)	__________Children/Child	
202	Have you ever had abortion or stillbirth? (Induced or non-induced abortion)	1. Yes<br>2. No -------------------------→	204
203	If yes, how many?	________ Abortions/Stillbirths	
204	How old is your last child? (in months)	-------------	
205	When did you have your last normal menstrual period?	1. ___/____/____ (dd/mm/yyyy)<br>2. I don't know/Not sure	
206	How many months pregnant are you?	1. ____________ months<br>2. I don't remember/Not sure	
207	Is the pregnancy planned?	1. Yes 2. No	
208	In your current pregnancy, did you receive ANC services?	1. Yes<br>2. No	
**3. At nine months of pregnancy (G.A. of pregnancy)**			
209	When was your first ANC visit?	………DD/ ………MM.	
210	How many ANC visits have you received? (in number)	_______	
211	When you went for ANC visit, are you decided jointly with your husband?	1. Yes -----------------------→<br>2. No	301
212	If not, who made the last decision?	1. Wife<br>2. Husband<br>3. Other specify …………	

**PHASE II - INTERVIEW OF PREGNANT WOMEN, 3 TO 5 DAYS AFTER DELIVERY**			
**4. Women's utilization of and husbands involvement in ANC services**			
301	During your ANC visits, have you get enough information about delivery care and PNC services?	1. Yes<br>2. No	
302	Was your husband escort you to health facility during ANC visit?	1. Yes<br>2. No -------------------------→	304
303	Was he attend the health information with you at health facility?	1. Yes<br>2. No ---------------------→	305
304	If no for Q.No.302, what was the reason?	--------	
305	If no for Q.No.303, what was the reason?	------------	
306	Were you get regular ANC services? If not, what was the reason?	------------	

**5. Husbands' involvement in delivery care**			
401	When are you delivered your last child?	…/…/…./ (dd/mm/yyyy)	
402	Where are you gave birth your last child?	1. Health facility -------→<br>2. Home<br>3. Other specify _________	403
403	Who was attend your birth?	____________	
404	If at home; why?	……………………….	
405	Were you made a joint decision for your delivery place?	1. Yes --------------→<br>2. No<br>3. I don't remember	406
406	If not, who made the final decision?	1. Wife<br>2. Husband<br>3. Other specify ………….	
407	If you delivered at HF; was your husband escort you for labor and delivery to the health facility?	1. Yes<br>2. No ----------------→	409 & 410
408	If yes, was your husband enter and attend your labour and delivery in the room?	1. Yes ---------------→<br>2. No	501
409	If 'no' for Q.No. 407, why?	………………………	410
410	If 'no' for Q.No. 406, why?	………………………	
411	Who escorted you during your labour and delivery to the health facility?	1. My mother<br>2. Relatives<br>3. Neighbor<br>4. Other specify ……	

**PHASE III - INTERVIEW OF PREGNANT WOMEN, AT PNC PERIODS** (AT 6TH WKS AFTER DELIVERY)			
**5. Husbands' involvement in postnatal care**			
501	Were you get PNC service in your last childbirth?	1. Yes<br>2. No ----------------→	510
502	If yes, when was your 1st PNC visit?	……………………..dd/mm/yyyy	
503	How many PNC visits are you received? (number/count)	----------------------	
504	Was your husband escort you during PNC visit?	1. Yes<br>2. No ----------------→	507
505	Was your husband attend PNC information with you in the health unit?	1. Yes ----------------→<br>2. No	508
506	If no, for 'Q.No.505', what was the reason?	-------------	
507	If no, for 'Q.No.504', what was the reason?	------------	
508	Were you made a joint decision for your PNC services?	1. Yes ---------------→<br>2. No	601
509	If no, who made the final decision?	1. Wife<br>2. Husband<br>3. Relatives<br>4. Other specify ……	
510	If 'no' for Q.No. 501, what was the reason?	---------------	
